# Ruminal and Postruminal Digestibility Parameters of Locally Produced Non-GMO Full-Fat Soybeans, Extruded Full-Fat Soybeans and Soybean Cake in Cattle

**DOI:** 10.3390/ani16111583

**Published:** 2026-05-23

**Authors:** Bogdan Śliwiński, Kamil Witaszek, Jakub Kostecki

**Affiliations:** 1 Department of Animal Nutrition and Feed Science, National Research Institute of Animal Production, 44 Jurajska St., Aleksandrowice, 32-084 Morawica, Poland; bogdan.sliwinski@iz.edu.pl; 2 Department of Biosystems Engineering, Poznan University of Life Sciences, 50 Wojska Polskiego St., 60-637 Poznań, Poland; 3 Institute of Environmental Engineering, University of Zielona Góra, 65-417 Zielona Góra, Poland

**Keywords:** processing, raw soybeans, ruminal degradation, intestinal digestibility

## Abstract

The aim of this study was to characterize ruminal degradation and postruminal digestibility of dry matter (DM) and crude protein (CP) in full-fat raw soybeans (ffSB), extruded full-fat raw soybeans (effSB) and soybean cake (SBc) derived from three non-GMO locally grown soybean varieties (PETRINA, ERICA and VIOLA). To hasten data interpretation, ruminal degradation and postruminal digestibility of the conventional solvent-extracted soybean meal (SBM) was also investigated. Effective rumen degradation (ERD) of DM was lower for SBc (0.726) than for SBM (0.777; *p* < 0.001). Independent of soy variety, it was less for SBc than ffSB (0.801) and the least for effSB (0.783; *p* < 0.001). Intestinal DM digestibility was higher for SBM (0.946) compared to ffSB (0.708) and effSB (0.604), and regardless of soybean variety, it was lower for effSB than for SBc (0.866; *p* < 0.01). The ERD of CP was higher for ffSB (0.817) compared to SBM (0.6858; *p* = 0.007), and, independent of soy variety, it was less for SBC (0.773) than ffSB and the lowest for effSB (0.750; *p* < 0.001). Intestinal digestibility of CP was higher for SBM (0.998) compared to ffSB (0.944), effSB (0.926), and SBc (0.943). Regardless of soybean variety, it was lower for effSB than for ffSB and SBc (*p* < 0.002). However, interactions between product type and soybean variety were also detected for almost all investigated parameters, except for c for DM and c and ERD for CP (*p* < 0.001), with variety ERICA showing the lowest ERD and variety PETRINA showing the highest intestinal digestibility. The study demonstrated that the type of soybean processing and soybean variety significantly affected ERD and intestinal digestibility of DM and CP. Soy products (effSB and SBc) produced by the farmer on his own farm from non-GMO soybeans harvested on his own farm can serve as valuable feed material for cattle, making him independent from the need to purchase imported SBM.

## Simple Summary

This study examined how various processing methods for local, non-genetically modified soybeans affect nutrient digestion in cattle. While soybeans are a staple protein source in animal feed, many European farms remain reliant on imported soybean meal. Our research compared raw soybeans, extruded soybeans, soybean cake, and commercial soybean meal derived from three Polish varieties. The study specifically evaluated rumen and intestinal digestibility. The results revealed that extrusion and oil pressing significantly altered the digestion of protein and dry matter. Specifically, processed soybean products reduced protein breakdown in the rumen, though in some instances, they also lowered intestinal digestibility. Results also varied by soybean variety, suggesting that both genetics and processing conditions are key factors in feed quality. While commercial meal showed the highest intestinal digestibility, locally produced alternatives demonstrated strong nutritional potential. These findings suggest that local non-genetically modified soybeans can partially replace imported feeds, helping farmers become more self-sufficient, supporting local agriculture, and reducing the environmental footprint of long-distance transport.

## 1. Introduction

ffSB and, particularly, SBM are commonly used in ruminant nutrition [1,2,3,4,5,6]. Over many years, these feed materials were imported to the EU, mainly from Argentina, Brazil, USA, Paraguay and Ukraine, and thus were derived from genetically modified (GMO) soybean [3]. However, the area planted with soybeans in Europe has increased since 2012. This crop has been integrated into European crop rotations, reaching over one million hectares in EU in 2023, and financial support and subsidies combined with national policies are used to stimulate soybean cultivation in Europe [7]. This trend is further justified due to observed climate changes, resulting in environmental conditions becoming more favorable for soybean cultivation. It is also important to note that the need to transport imported ffSB and SBM increases the carbon footprint [8].

While SBM is the most commonly used feed material derived from ffSB in ruminant nutrition, other materials can also be used as valuable sources of protein, energy or both. These include whole ffSB [9,10,11], roasted ffSB [1,6,12] and extruded or expelled SBM [3,6].

Because the area of soybean cultivation in numerous countries in the EU is still limited, such as in Poland, the availability of material for large industrial plants that process large amounts of seeds is limited [13]. Consequently, the development of local processing of soybeans on a small scale is observed, which does not require large batches of feed material, and thus, material can be processed by the producer on its own after investment in the necessary equipment for on-farm soybean processing. Due to the low cost of the mentioned investment, soybean extrusion is getting increasingly popular. In addition, extrusion allows for obtaining feed material with reduced concentration of antinutritional factors [13,14], which is especially important in the case of monogastrics nutrition but is of no great importance for ruminants—it may affect rumen degradation of protein and postruminal protein digestibility [1], which is important with regard to ruminant nutrition. Furthermore, effSB are often pressed to ‘remove’ oil, resulting in SBc, or more specifically, SBc derived from effSB. However, knowledge on the nutritive value of feed materials derived from non-GMO locally grown soybean for ruminants is limited.

Because feed material derived from soybeans are considered a predominantly valuable source of protein in diets for ruminants, the aim of this study was to better characterize ruminal degradation and postruminal digestibility of protein in effSB and SBc derived from effSB. It was hypothesized that the thermobaric processing of ffSB, i.e., extrusion and subsequent oil pressing, would affect ruminal degradability and postruminal digestibility of protein.

## 2. Materials and Methods

### 2.1. Soybean Feed Material Preparation

Feed material used for the study was described in detail in Świątkiewicz et al. [13]. Briefly, soybeans from three soybean (*Glycine max* (L.) *Merr.*) varieties, that is ERICA, PETRINA and VIOLA (DANKO Hodowla Roślin sp. z o.o., 27, Choryń, 64-000 Kościan, Poland), recommended for cultivation in the climate and soil conditions of northeastern Europe, were used. These varieties were selected to represent early, late, and very late varieties [15].

ffSB were subjected to thermobaric treatment (extrudate) and oil pressing (SBc/press cake). ffSB were processed using an E-75 extruder (AgroFeedingTech, Poznań, Poland; four working chambers with PT-100 temperature sensors; year of manufacture 2019; drive motor power 7.5 kW; L/D ratio 8.5:1; single screw, screw rotational speed 480 rpm, die nozzle diameter 8 mm, pressure up to 19–21 MPa, temperature range 40–200 °C, time of exposure approx. 30–60 s, yield 50–75 kg·h^−1^). The extruded material was dried with a 2.2 kW conveyor equipped with a water vapor extractor (1.1 kW fan, PT-100 sensors at inlet and outlet), then pressed to extract oil using a PS-60 screw press (AgroFeedingTech, Poznań, Poland; year of manufacture 2020; drive motor power 7.5 kW; screw rotational speed 42 rpm; exit slot 7 mm; yield 40–50 kg·h^−1^) and the resulting SBc was ground using an RUD2-16 roller mill (Spomasz, Ostrów Wielkopolski, Poland; year of manufacture 1984; drive motor power 4 kW; working slot 2 mm; yield 40–50 kg·h^−1^). During the extrusion process of the tested soybeans, the average temperature in the first chamber of the extruder was 92.9 °C with fluctuations from 90.1 °C to 95.3 °C; in the second chamber, the average temperature was 110 °C with fluctuations from 107.3 °C to 115.4 °C; in the third chamber, the average temperature was 128.6 °C with fluctuations from 123.9 °C to 133.5 °C; in the fourth chamber, the average temperature was 127.5 °C with fluctuations from 124.8 °C to 130.2 °C. Moreover, commercially available SBM derived from transgenic soy varieties was included as additional feed material to hasten data interpretation. The variety of soy from which the SBM was derived was unknown.

### 2.2. Animal Experiment—In Sacco Method

The Rumen degradability and intestinal digestibility of DM and CP of ffSB, effSB, SBc and SBM were determined using the *in sacco* method on 3 adult non-lactating cows (heifers; 700 ± 80 kg) fitted with ruminal and duodenal T type cannula. The cows were fed a standard diet consisting of 7.9 kg of hay and 2.6 kg of a feed mixture consisting of barley meal, SBM, wheat bran, mineral and vitamin supplement and salt (46.5, 30, 20, 3 and 0.5% in DM, respectively). The inclusion of soybean meal would not affect the results—if there were any effect, it would be present in all samples across the different treatments. Cows were fed two times a day (7:00 a.m. and 3:00 p.m.).

Polyester bags with a pore size of 50 µm and dimensions of 10 × 5 cm (R510) and 5 × 5 cm (R55) (ANKOM Technology Corporation, Fairport, NY, USA) pretreated with acetone were used for ruminal incubation and intestinal digestibility, respectively. Bags were filled with 2.3 g of the sample for rumen degradation analysis and 1 g of the sample for of intestinal digestibility analysis (20 mg of DM sample/cm^2^ of the surface of the bag; Michalet-Doreau et al. [16]). Prior to analysis, feeds were ground to pass 1.5 mm.

To assess rumen degradation, bags were incubated in the rumen for 0, 2, 4, 8, 24, and 48 h. All bags, except those for the 16 h incubation period, were simultaneously placed in the rumen and sequentially removed after 2, 4, 8, 24, and 48 h. When the bags were removed after 8 h, bags incubated in the rumen for 16 h were placed in the rumen and removed the next day along with the 24 h bags [17,18] (Micek 2008, Beak and Choi 2017). All samples were incubated in one run. For each feed and incubation time of 2, 4, and 8 h, 3 bags per cow were placed in the rumen; for the 16 and 24 h periods—4 bags per cow; and for 48 h—5 bags per cow. Once removed from the rumen, bags were rinsed with cold tap water and frozen (−20 °C). Bags not incubated in the rumen (time “0” of incubation) were soaked in lukewarm water (39 ± 1 °C) for 30 min and then also frozen. Subsequently, bags were thawed, washed in cold water (two cycles in a washing machine without centrifugation, 10 min each), and dried in a forced-air oven at 50 °C for 48 h. The residues from each bag, time, and cow were composited to yield one sample.

To assess intestinal digestibility, bags were incubated for 16 h in the rumen, then incubated at 39 °C for 2.5 h in pepsin–HCl solution, as described in detail in Rajtar et al. [19]. After rinsing with tap water, bags were placed through the duodenal cannula into the small intestine and recovered in the feces. Only bags that were recovered in feces within 24 h after insertion of the intestine were used for further analysis. Intestinal digestibility was run in four runs.

Effective rumen degradability (ERD) was calculated as proposed by Ørskov–McDonald [20] using the following formula:

ERD = a + ((b × k)/(k + c))(1)

where

a—soluble fraction immediately degraded in the rumen (%).
b—fraction degraded in the rumen at rate k (%).
k—rate of rumen degradability of fraction b (%/h).
c—rate of outflow from the rumen 0.05/h.

Furthermore, rumen DM and CP degradation at particular time points were calculated as follows:

Degradation at time t = weight of residue after t hours of ruminal incubation × DM of the material/initial weight of the sample × DM of the material

Intestinal digestibility (ID) was calculated using the following formula:

ID DM = ((DM × (100 − Dg16) − (DM × (100 − DgI DM))/(DM × (100 − Dg16))(2)

where

DM = amount of dry matter in the sample before incubation (g).
Dg16 = dry matter degradability in the rumen after 16 h of incubation (%).
DgI = dry matter digestibility of dry matter throughout the gastrointestinal tract (%).

Total digestibility (TTD) was calculated as the difference between the component content in the starting material and that in the material recovered from the mobile bags.

The chemical compositions of the feeds and feed residues were analyzed according to the AOAC methods [21] as described in Świątkiewicz et al. [13] (where you can also find the results of analyses of antinutritional substances and mycotoxins). Pooled samples from individual incubation times for each sample were analyzed for DM and CP as described for feeds.

### 2.3. Statistical Analysis

Data were analyzed using the MIXED procedure of SAS (version 9.4; SAS Institute Inc., Cary, NC, USA). The experiment was arranged as a 3 × 3 factorial design (soybean processing × soybean variety) with an additional control treatment (SBM), and therefore analyses were conducted in two steps. In the first step, all treatments were included in the model, with treatment as a fixed effect and cow as a random effect. When the treatment effect was significant (group, *p* < 0.05), contrasts were used to compare the control with the remaining treatments. In the second step, only the nine soybean treatments were analyzed to evaluate the effects of processing, variety, and their interaction. Processing method, soybean variety, and their interaction were included as fixed effects, and cow was considered a random effect. When significant effects were detected (*p* ≤ 0.05), means were separated using Tukey–Kramer adjustment. Normality of residuals was assessed using PROC UNIVARIATE prior to analysis.

## 3. Results

The chemical composition of experimental feeds is presented in Table 1.

CP concentration in ffSB and effSB was comparable. However, EE concentration was higher and CF concentration was lower for effSB compared to ffSB. Compared to ffSB and effSB, CP concentration in SBc, and especially SBM, was greater; in contrast, EE concentration and CF concentrations were lower. EE concentration in ffSB was the lowest for the ERICA variety, whereas CP concentration in SBc derived from the PETRINA variety was higher and comparable to that of SBc derived from the ERICA and VIOLA varieties.

Estimated rumen solubility of DM (parameter “a”) was lower for SBM compared to ffSB, effSB and SBc (effect of group, *p* < 0.001; Table 2).

Independent of soy variety, it was lower for ffSB than for SBc and effSB (effect of product, *p* < 0.001); however, interaction between the effect of product and variety of soybean was also observed (*p* < 0.001); estimated rumen solubility of DM (parameter “a”) was the highest for ffSB derived from VIOLA and especially PETRINA variety. Potentially degradable fraction “b” of DM differed between SBM and SBc (effect of group, *p* < 0.001) but not between SBM and ffSB and effSB. Independent of soy variety, it was the highest for SBc but did not differ between ffSB and effSB (effect of product, *p* < 0.001). However, for this parameter an interaction between the effect of product and variety was also detected (*p* = 0.004), due to the mentioned highest potentially degradable fraction “b” of DM (parameter “c”) for SBc derived from variety PETRINA and VIOLA. The parameter “c” DM was higher in SBM compared to ffSB, effSBs and SBc (effect of group, *p* < 0.001). It was lower for SBc than ffSB and effSB (effect of product, *p* < 0.001), and was higher for variety ERICA than other varieties (effect of variety, *p* = 0.013). ERD of DM was lower for SBc than SBM (effect of group, *p* < 0.001). Independent of soy variety, it was lower for SBc than ffSB and the least for effSBs (effect of product, *p* < 0.001). However, interaction between main effects was detected (*p* < 0.01). ERD of DM for SBc derived from PETRINA was the lowest, while ERD of DM for ffSB from this variety was the highest. In general, DM degraded faster for SBM and particularly SBc than in ffSB and effSB.

Intestinal DM digestibility was higher for SBM compared to ffSB and effSB (group effect, *p* < 0.001; Table 2). Regardless of soybean variety, it was lower for effSB than for SBc (product effect, *p* < 0.01); however, an interaction between product and soybean variety was also observed (*p* < 0.001); intestinal DM digestibility was highest for SBc derived from the VIOLA variety and lowest for effSM derived from the ERICA variety.

Total tract DM digestibility was higher for SBM compared to ffSB of the PETRINA variety and effSB (group effect, *p* < 0.001; Table 2). Regardless of the soybean variety, it was lower for effSB than for SBc and ffSB (product effect, *p* < 0.01) and also between the ERICA variety and PETRINA and VIOLA; however, an interaction between the effect of the product and the soybean variety was also observed (*p* < 0.001); total DM digestibility was the highest for SBc from the VIOLA variety and the lowest for the effSB from ERICA.

Estimated rumen solubility of CP (parameter “a”) was lower for SBM compared to ffSB, effSB and SBc (effect of group, *p* < 0.001; Table 3).

Independent of soy variety, it was lower for effSB than SBc, and lower for SBc than ffSB (effect of product, *p* < 0.001). However, for this parameter an interaction between the effect of product and variety was also detected (*p* < 0.001), due to the highest soluble fraction of CP (parameter “a”) observed for ffSB and varieties PETRINA and VIOLA and the lowest for effSB and variety VIOLA. Potentially degradable fraction of CP (parameter “b”) was lower for SBc derived from variety PETRINA and ERICA, and ffSB derived from ERICA, compared to SBM (effect of group, *p* = 0.009). Significant effect of product was shown for this parameter (*p* = 0.017), but no differences between products were detected by post hoc test. On the other hand, the potentially degradable fraction of CP (parameter “b”) was higher for variety VIOLA than for variety PETRINA (effect of variety, *p* = 0.040). The CP degradation rate of the fraction b (parameter “c”) was higher in SBM compared to ffSB, effSB and SBc (effect of group, *p* < 0.001), but was not affected by product type and variety (*p* ≥ 0.103). ERD of CP was higher for ffSB compared to SBM (effect of group, *p* = 0.007). Independent of soy variety, it was lower for SBc than ffSB and the lowest for effSB (effect of product, *p* < 0.001). However, interaction between main effect was detected (*p* < 0.001), due to the highest ERD of CP for ffSB derived from the PETRINA variety and the least for effSB derived from the VIOLA variety (*p* < 0.001).

Intestinal digestibility of crude protein was higher for SBM compared to ffSB, effSB, and SBc, except SBc from the VIOLA variety (group effect, *p* < 0.001; Table 2). Regardless of soybean variety, it was lower for effSB than for ffSB and SBc (product effect, *p* < 0.002); however, an interaction between product and soybean variety was also observed (*p* < 0.001); intestinal digestibility of CP was highest for SBc from the VIOLA variety and lowest for the ERICA variety.

Total digestibility of CP was higher for SBM compared to effSB from any of the tested varieties and ERICA and PETRINA SBc (group effect, *p* < 0.001; Table 2). Regardless of the soybean variety, it was lower for effSB compared to SBc and ffSB (product effect, *p* < 0.01); however, an interaction between the effect of product and soybean variety was also observed (*p* < 0.001); the total digestibility of CP was highest for SBc derived from the VIOLA variety and lowest for the VIOLA effSB.

**Figure 1:**
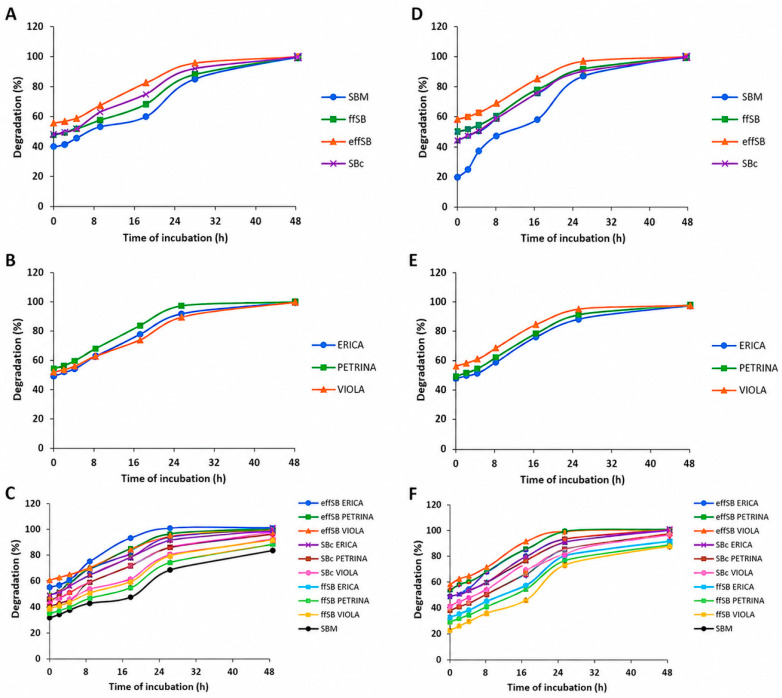
Degradation of DM after 2, 4, 8, 16, 24 and 48 h incubation in the rumen (effect of product—(**A**), effect of soy variety—(**B**) and all experimental variants—(**C**) and degradation of CP (effect of product—(**D**), effect of soy variety—(**E**) and all experimental variants—(**F**)).

Dynamic of DM degradation over time is presented in Figure 1A–C.

Degradation for ffSB was the highest during the initial step of incubation (especially for variety PETRINA, Figure 1A,C), as reflected by high content of the easily degradable fraction (Table 2). On the other hand, SBM had the lowest initial degradation, whereas effSB and SBc were intermediate. Of the four investigated products, SBM and SBc degraded more slowly over 24 h of incubation (Figure 1A).

Dynamic of CP degradation over time is presented in Figure 1D–F. CP of ffSB degraded the fastest, that of SBM the slowest, whereas effSB and SBc were characterized by an intermediate dynamic of CP degradation (Figure 1D). Of the varieties, PETRINA showed the most dynamic CP degradation in the initial stages of incubation in the rumen, as reflected by high content of the easily degradable fraction (Table 3).

## 4. Discussion

The aim of this study was to better characterize ruminal degradation and postruminal digestibility of DM and CP in ffSB, effSB and SBc derived from three non-GMO soy varieties grown in Poland. Based on the widely known impact of heat treatment on protein degradation in the rumen, and also postruminal protein digestion [22,23], it was expected that soybean thermobaric processing, i.e., extrusion, would reduce ruminal degradability of protein as well as affect its postruminal digestibility. Results of the study confirmed this hypothesis. However, some parameters of ruminal and postruminal digestibility differed compared to ‘conventional’ SBM.

The CP content in ffSB ranged from 360 to 390 g/kg DM. Thus, protein concentration in soybeans was slightly lower than the content reported in soybeans derived from plants and varieties grown in regions with climate conditions more favorable for soy. EE content was similar to that reported for ffSB, but CF concentration was higher than typically reported for soybeans and ranged from 70 to 90 g/kg DM [24,25,26,27,28]. Those differences in the chemical composition of soybeans derived from locally grown soy can affect the final results of their processing, justifying the current study.

As expected, SBM had a higher concentration of protein and the lowest content of fat, due to a very efficient removal of fat from soybeans. As also expected, fat concentration in SBc was substantially reduced compared to ffSB, and was in a range typical for this type of feed [24,28]. On the other hand, fiber concentration, unexpectedly, was substantially less in effSB compared to ffSB. This observation is difficult to explain, but others also noticed reduced fiber concentration in extruded full-fat soybeans, particularly when high temperature of treatment was used [27]. However, it cannot be excluded that high fat content in ffSB in combination with fat ‘structure’ could contribute to lower precision of fiber analysis and overestimation of fiber concentration [28].

The lower ERD observed for effSB and SBc compared with ffSB can be attributed primarily to processing-induced changes in protein structure and lipid content. High temperature and pressure during extrusion promote protein denaturation and Maillard-type reactions, which are associated with reduced ruminal protein degradability and an increased proportion of rumen-undegraded protein [29]. However, ADIN (acid detergent insoluble nitrogen), an indicator for Maillard reaction, was not marked in our experiment. Similarly, oil pressing (cake production) removes part of the easily degradable soluble fraction and exposes proteins to moderate heat, decreasing their solubility and susceptibility to enzymatic breakdown [30]. In contrast, ffSBs retain native, highly degradable proteins and soluble components, resulting in higher ruminal degradability values for both DM and CP. As already mentioned, lower ERD of DM and CP in effSB and SBc were expected.

The ERD of DM and CP in ffSB and SBM was within typical reported values for those feeds, that is ~0.801 for DM and ~0.817 for CP for ffSB and ~0.777 for DM and ~0.686 for CP for SBM [27]. In terms of high-producing ruminants, bypass protein concentration in high-protein feeds is especially important. This was not different between SBM and effSB and SBM and SBc, indicating that extrusion parameters used in the current study did not allow for efficient CP protection from degradation in the rumen. Despite the fact that the content of the soluble fraction of CP was greater in effSB and SBc than in SBM, the degradation rate of the potentially degradable fraction was lower, resulting in similar ERD of CP among those three feed materials. However, it is worth mentioning that, in general, differences in ERD of CP between ffSB and effSB and ffSB and SBc were not substantial (from 0.817 to 0.750 and 0.773 for ffSB, effSB and SBc, respectively). In particular, the temperature of treatment and the time of exposure to the increased temperature are the most important factors affecting CP protection from degradation in the rumen [2,31]. Nowak et al. [27] have shown that exposure of soybean temperature of 145 °C or greater substantially reduced ERD of CP. Thus, it can be concluded that the temperature applied in the current study or the time the feed material was exposed to this temperature were too low to reduce ruminal degradation of DM and CP substantially. This is supported by reported substantial concentrations of antinutritional factors in investigated feeds, including trypsin inhibitor [13].

While the product itself had an impact on the ruminal digestion of the investigated feeds, the effect of the soy variety was also significant for some of the investigated parameters. Of importance, there were interactions between the effect of the variety and the effect of the product. Those interactions can be partially attributed to differences in chemical composition between varieties. However, independent of those interactions, in some instances, the effect of variety had a higher impact on the ERD of DM or CP than the processing of the feed material. This mainly applied to the ruminal degradation of DM. When it comes to the ERD of CP, it was not affected by the interaction between plant variety and soybean processing. Consequently, even though the origin of soybeans, that is, the variety of the plant, can affect degradation constants, in general, it has no substantial impact on CP degradation in the rumen. Thus, the processing of the material and the parameters of thermobaric processing should be considered the most important factors affecting ruminal degradability of soy protein.

The intestinal digestibility of DM and CP varied significantly between products and, to a lesser extent, between varieties. Generally, SBc showed the highest intestinal digestibility of DM and high CP digestibility, while effSB had the lowest intestinal DM digestibility, indicating that heat treatment during extrusion may have partially reduced intestinal availability. This reduction is likely linked to Maillard reactions and protein denaturation, which decrease the enzymatic accessibility of protein fractions in the small intestine despite lowering ruminal degradability. However, given that extrusion did result in a substantial reduction in DM and, especially, CP degradation in the rumen, it is unlikely that CP was overprotected. Most likely, fat present in the material affected its intestinal digestibility. High fat concentration in full-fat soybeans can reduce in situ ruminal degradation because the fat forms a coating that physically protects the protein and other nutrients from rumen microbes. This lipid coating can decrease the rate and extent of DM and protein disappearance from the bags, making more of the protein bypassing the rumen [32,33,34]. According to Rossi et al. [35] and Alizadeh et al. [36] the mechanisms of reduced degradation include physical protection, reduced solubility, and reduced degradation rate. High fat concentrations create a lipid matrix that physically coats the soybean particles, protecting protein and other nutrients from microbial access and enzymatic degradation in the rumen. Furthermore, the presence of fat reduces nitrogen solubility in the rumen. This occurs because the lipid coating makes the nitrogen fraction less accessible to ruminal microorganisms. Studies [35,36] show that this effect is noticeable even after short incubation times (e.g., 8 h) and results in a lower overall rate of degradation.

The observed varietal differences—with VIOLA generally showing higher postruminal CP digestibility—suggest that intrinsic protein structure and composition influence the degree to which processing affects intestinal digestion. Overall, the interaction between variety and product type suggests that both genetic and technological factors can together determine nutrient availability in the small intestine, unlike ruminal digestibility, underscoring the need for variety-specific optimization of processing conditions to preserve intestinal protein digestibility. However, of importance, intestinal digestibility of CP was greater for SBM than other products. This may be a result of the removal of antinutritional factors and other components during processing. Processing methods can improve digestibility by breaking down complex proteins and reducing the levels of antinutritional factors, which increases the amount of protein and amino acids that are digestible in the small intestine. As reported by the Busanello et al. [37] the greater intestinal digestibility of CP in ruminants fed SBM is likely due to a combination of factors, including its higher content of ruminally undegradable protein (RUP), which passes to the small intestine for digestion, and the absence of certain high-protein by-products found in alternative protein sources that can decrease overall feed digestibility. These factors lead to more protein being absorbed as amino acids in the small intestine compared to other protein products, which may have a more rumen-degradable protein profile or higher fiber content that impedes digestibility. SBM has a protein profile that is less likely to be broken down in the rumen. Unlike some alternative protein sources that contain high levels of fiber from by-products, SBM has a low fiber content. Furthermore, the heat treatments used to process SBM, while potentially reducing rumen degradability, increase the amount of protein that bypasses the rumen and is digestible in the small intestine, especially when heat is mild. However, severe heat can have negative effects on protein digestibility. However, total tract digestibility did not differ between treatments.

## 5. Conclusions

The present study demonstrated that both processing method and soybean variety significantly affect ruminal degradability and postruminal digestibility of DM and CP in locally produced non-GMO soybeans. Extrusion and subsequent oil pressing reduced ERD of both DM and CP compared with ffSB, confirming the impact of thermobaric processing on protein availability in the rumen. However, the magnitude of this reduction was moderate, indicating that the applied extrusion conditions were insufficient to achieve substantial protection of protein from ruminal degradation.

Despite the lower ERD, effSB and SBc did not consistently improve the overall availability of digestible protein due to reduced intestinal digestibility, particularly in effSB. This suggests that heat-induced changes in protein structure and lipid interactions may have limited enzymatic accessibility in the small intestine. In contrast, SBM exhibited the highest intestinal digestibility of both DM and CP, confirming its superior nutritional efficiency under the conditions of this study.

The results also revealed significant interactions between processing method and soybean variety, indicating that intrinsic compositional differences between cultivars can influence the response to technological treatment. However, the effect of processing was generally more pronounced than varietal differences, particularly for CP degradability.

Overall, effSB and SBc derived from locally produced non-GMO soybeans can serve as alternative feed materials for ruminants. Nevertheless, optimization of processing parameters, particularly temperature and exposure time, is required to improve ruminal protein protection without compromising intestinal digestibility.

## Figures and Tables

**Table 1:** Chemical composition of experimental feeds.

Product	Variety	DM (g/kg)	CA (g/kg DM)	CP (g/kg DM)	EE (g/kg DM)	CF (g/kg DM)
ffSB	ERICA	902.1	65.7	359.3	176.6	99.4
ffSB	PETRINA	876.4	61.3	389.9	201.6	72.7
ffSB	VIOLA	910.3	54.8	357.4	207.5	92.2
effSB	ERICA	937.9	60.6	360.9	229.9	47.9
effSB	PETRINA	935.3	57.8	400.8	216.9	43.9
effSB	VIOLA	944.9	59.9	346.3	243.3	48.2
SBc	ERICA	951.5	71.7	421.8	93.4	57.9
SBc	PETRINA	944.5	67.7	455.5	91.5	50.7
SBc	VIOLA	957.7	71.4	428.2	92.1	57.7
SBM	Unknown	887.1	77.4	511.0	13.0	51.5

ffSB—full-fat soybean; effSB—extruded full-fat soybean; SBc—soybean cake; SBM—soybean meal. Dry matter (DM); Crude ash (CA); crude protein (CP); Ether extract (EE); Crude fiber (CF).

**Table 2:** Effect of the product type (ffSB, effSB, SBc, or SBM) soybean variety on degradation constants (a, b and c), effective rumen degradation (ERD), intestinal and total tract digestibility of DM.

Product	Variety	Ruminal Degradability				Intestinal Digestibility	Total Tract Digestibility
		a	b	c	ERD		
ffSB		0.512 ^A^	0.577 ^B^	0.061 ^A^	0.801 ^A^	0.708 ^B^	0.963 ^A^
effSB		0.453 ^B^	0.591 ^B^	0.064 ^A^	0.783 ^B^	0.604 ^C^	0.927 ^B^
SBc		0.458 ^B^	0.794 ^A^	0.027 ^B^	0.726 ^C^	0.866 ^A^	0.957 ^A^
	ERICA	0.450 ^B^	0.625	0.062 ^A^	0.778	0.708	0.958 ^A^
	PETRINA	0.497 ^A^	0.679	0.044 ^B^	0.768	0.726	0.945 ^B^
	VIOLA	0.476 ^A^	0.659	0.047 ^AB^	0.764	0.744	0.946 ^B^
SBM		0.400	0.645	0.127	0.777	0.946	0.959
ffSB	ERICA	0.450 *^C^	0.580 ^BC^	0.071 *^A^	0.789 ^ABC^	0.733 *^CDE^	0.949 ^AB^
ffSB	PETRINA	0.577 *^A^	0.586 ^C^	0.061 *^AB^	0.827 ^A^	0.716 *^DE^	0.941 *^AB^
ffSB	VIOLA	0.510 *^B^	0.665 ^BC^	0.051 *^ABC^	0.786 ^BC^	0.874 ^ABC^	0.948 ^AB^
effSBs	ERICA	0.452 *^C^	0.579 ^BC^	0.080 *^A^	0.809 ^AB^	0.659 *^DE^	0.932 *^ABC^
effSBs	PETRINA	0.446 *^C^	0.619 ^BC^	0.052 *^ABC^	0.761 *^CD^	0.765 *^BCD^	0.916 *^C^
effSBs	VIOLA	0.461 *^C^	0.573 ^BC^	0.062 *^AB^	0.778 ^BC^	0.587 *^E^	0.882 *^D^
SBc	ERICA	0.448 *^C^	0.714 *^B^	0.034 *^BC^	0.735 *^DE^	0.933 ^A^	0.941 *^AB^
SBc	PETRINA	0.470 *^C^	0.933 *^A^	0.018 *^C^	0.715 *^E^	0.896 ^AB^	0.926 *^BC^
SBc	VIOLA	0.457 *^C^	0.738 *^AB^	0.029 *^BC^	0.727 *^DE^	0.969 ^A^	0.952 ^A^
SE		0.010	0.074	0.017	0.021	0.050	0.008
Group		<0.001	<0.001	<0.001	<0.003	<0.001	<0.001
Product		<0.001	<0.001	<0.001	<0.001	<0.01	<0.001
Variety		<0.001	0.291	0.013	0.108	0.429	0.003
Product × variety		<0.001	0.004	0.429	<0.001	<0.001	<0.001

ffSB—full-fat soybean; effSB—extruded full-fat soybean; SBc—soybean cake; SBM—soybean meal. a—soluble fraction; b—potentially degradable fraction; c—rate of degradation of fraction b; ERD—effective rumen degradability. Different uppercase letters in the same column indicate differences between treatments (*p* < 0.05) and asterisks indicate differences used for “Group” when each soybean feed material was treated as a separate group and compared to SBM (*p* < 0.05).

**Table 3:** Effect of the product type (ffSB, effSB, SBc, or SBM) soybean variety on degradation constants (a, b and c), effective rumen degradation (ERD), intestinal and total tract digestibility of CP.

Product	Variety	Ruminal Degradability				Intestinal Digestibility	Total Tract Digestibility
		a	b	c	ERD		
ffSB		0.541 ^A^	0.554 ^B^	0.060	0.817 ^A^	0.944 ^A^	0.995 ^A^
effSB		0.402 ^C^	0.671 ^B^	0.054	0.750 ^C^	0.926 ^B^	0.986 ^B^
SBc		0.465 ^B^	0.598 ^AB^	0.053	0.773 ^B^	0.943 ^A^	0.994 ^A^
	ERICA	0.442 ^B^	0.619 ^AB^	0.062	0.784 ^A^	0.9185	0.992
	PETRINA	0.515 ^A^	0.552 ^B^	0.053	0.792 ^B^	0.9394	0.992
	VIOLA	0.452 ^B^	0.652 ^A^	0.045	0.765 ^AB^	0.9558	0.991
SBM		0.211	0.7695	0.103	0.6858	0.998	0.993
ffSB	ERICA	0.460 *^D^	0.584 *^AB^	0.071 *	0.801 *^B^	0.924 *^E^	0.990 ^ABC^
ffSB	PETRINA	0.619 *^A^	0.552 *^B^	0.065 *	0.852 *^A^	0.954 *^CD^	0.991 ^AB^
ffSB	VIOLA	0.543 *^B^	0.650 ^AB^	0.045 *	0.798 *^B^	0.987 ^A^	0.993 ^AB^
effSB	ERICA	0.423 *^F^	0.654 ^A^	0.059 *	0.777 ^BC^	0.924 *^DE^	0.986 *^CD^
effSB	PETRINA	0.426 *^EF^	0.659 ^A^	0.048 *	0.749 ^CD^	0.957 *^BC^	0.985 *^D^
effSB	VIOLA	0.358 *^G^	0.701 ^A^	0.055 *	0.725 ^D^	0.941 *^CDE^	0.978 *^E^
SBc	ERICA	0.443 *^DEF^	0.621 ^AB^	0.057 *	0.773 ^BC^	0.931 *^CDE^	0.990 *^BCD^
SBc	PETRINA	0.500 *^C^	0.569 *^AB^	0.047 *	0.774 ^BC^	0.940 *^CDE^	0.989 *^BCD^
SBc	VIOLA	0.453 *^DE^	0.605 ^AB^	0.055 *	0.770 ^BC^	0.983 ^AB^	0.994 ^A^
SE		0.010	0.028	0.008	0.073	0.009	0.002
Group		<0.001	<0.001	0.009	0.001	<0.001	<0.001
Product		<0.001	<0.001	0.017	0.312	0.002	<0.001
Variety		<0.001	<0.001	0.040	0.103	<0.001	0.867
Product × variety		<0.001	<0.001	0.205	0.150	<0.001	<0.001

ffSB—full-fat soybean; effSB—extruded full-fat soybean; SBc—soybean cake; SBM—soybean meal. a—soluble fraction; b—potentially degradable fraction; c—rate of degradation of fraction b; ERD—effective rumen degradability. Different uppercase letters in the same column indicate differences between treatments (*p* < 0.05) and asterisks indicate differences used for “Group” when each soybean feed material was treated as a separate group and compared to SBM (*p* < 0.05).

## Data Availability

The original contributions presented in this study are included in the article. Further inquiries can be directed to the corresponding authors.

## Abbreviations

The following abbreviations are used in this manuscript:

GMO	Genetically Modified Organism
non-GMO	non-Genetically Modified Organism
CAP	Common Agricultural Policy
AOAC	Association of Official Analytical Chemists
ERD	Effective Rumen Degradability
DM	Dry Matter
CP	Crude Protein
RUP	Rumen Undegradable Protein

## References

[1] Stern M.O., Santos K.A., Satter L.D. (1985). Protein Degradation in Rumen and Amino Acid Absorption in Small Intestine of Lactating Dairy Cattle Fed Heat-Treated Whole Soybeans. J. Dairy Sci..

[2] Faldet M.A., Son Y.S., Satter L.D. (1992). Chemical, In Vitro, and In Vivo Evaluation of Soybeans Heat-Treated by Various Processing Methods. J. Dairy Sci..

[3] Vindis P., Mursec B., Janzekovic M., Cus F. (2007). Processing of Soybean Meal into Concentrates and Testing for Genetically Modified Organism (GMO). J. Achiev. Mater. Manuf. Eng..

[4] Grummer R.R., Luck M.L., Barmore J.A. (1994). Lactational Performance of Dairy Cows Fed Raw Soybeans, with or Without Animal By-Product Proteins, or Roasted Soybeans. J. Dairy Sci..

[5] van Dijk H.J., O’Dell G.D., Perry P.R., Grimes L.W. (1983). Extruded Versus Raw Ground Soybeans for Dairy Cows in Early Lactation. J. Dairy Sci..

[6] Scott T.A., Combs D.K., Grummer R.R. (1991). Effects of Roasting, Extrusion, and Particle Size on the Feeding Value of Soybeans for Dairy Cows. J. Dairy Sci..

[7] Román A. (2023). EU Protein Strategy.

[8] De Boer H.C., Van Krimpen M.M., Blonk H., Tyszler M. (2014). Replacement of Soybean Meal in Compound Feed by European Protein Sources Effects on Carbon Footprint.

[9] Banta J.P., Lalman D.L., Krehbiel C.R., Wettemann R.P. (2008). Whole Soybean Supplementation and Cow Age Class: Effects on Intake, Digestion, Performance, and Reproduction of Beef Cows1. J. Anim. Sci..

[10] Vicenti A., Toteda F., Di Turi L., Cocca C., Perrucci M., Melodia L., Ragni M. (2009). Use of Sweet Lupin (*Lupinus Albus* L. Var. Multitalia) in Feeding for Podolian Young Bulls and Influence on Productive Performances and Meat Quality Traits. Meat Sci..

[11] Barletta R.V., Gandra J.R., Freitas Junior J.E., Verdurico L.C., Mingoti R.D., Bettero V.P., Benevento B.C., Vilela F.G., Rennó F.P. (2016). High Levels of Whole Raw Soya Beans in Dairy Cow Diets: Digestibility and Animal Performance. J. Anim. Physiol. Anim. Nutr..

[12] Golshan S., Pirmohammadi R., Khalilvandi-Behroozyar H. (2019). Microwave Irradiation of Whole Soybeans in Ruminant Nutrition: Protein and Carbohydrate Metabolism In Vitro and in Situ. Vet. Res. Forum.

[13] Świątkiewicz M., Witaszek K., Sosin E., Pilarski K., Szymczyk B., Durczak K. (2021). The Nutritional Value and Safety of Genetically Unmodified Soybeans and Soybean Feed Products in the Nutrition of Farm Animals. Agronomy.

[14] Świątkiewicz M., Szczepanik K., Gala Ł., Grela E.R., Witaszek K., Barszcz M., Tuśnio A., Taciak M. (2024). Determination of the Impact of Extruded Soybean Press Cake on Rearing and Health Indices of Piglets. Agriculture.

[15] COBORU (2024). Wyniki Doświadczeń Odmianowych Soi i Roślin Bobowatych Grubonasiennych 2021–2023.

[16] Michalet-Doreau B. (1992). Aliments Concentrés Pour Ruminants: Dégradabilité in Situ de l’azote Dans Le Rumen. INRAE Prod. Anim..

[17] Micek P. (2008). Przydatność żywieniowa ziarna krajowych gatunków i odmian zbóż dla przeżuwaczy. Zeszyty Naukowe Uniwersytetu Rolniczego im, Hugona Kołłątaja w Krakowie, Rozprawy nr 326.

[18] Baek Y.C., Choi H. (2017). Evaluation of non-conventional feeds for ruminants using in situ nylon bag and the mobile bag technique. J. Korea Acad.-Ind. Coop. Soc..

[19] Rajtar P., Górka P., Schwarz T., Micek P. (2020). Effect of hybrid rye and maize grain processing on ruminal and postruminal digestibility parameters. Ann. Anim. Sci..

[20] Ørskov E.R., McDonald I. (1979). The estimation of protein degradability in the rumen from incubation measurements weighed according to rate of passage. J. Agric. Sci..

[21] AOAC (2005). Official Methods of Analysis.

[22] Paya H., Taghizadeh A., Hosseinkhani A., Mohammadzadeh H., Janmohammadi H., Moghaddam G. (2022). Effects of Different Heat Processing Methods of Rapeseed on Ruminal and Post-Ruminal Nutrient Disappearance. J. Hell. Vet. Med. Soc..

[23] Karlsson L., Ruiz-Moreno M., Stern M.D., Martinsson K. (2012). Effects of Temperature during Moist Heat Treatment on Ruminal Degradability and Intestinal Digestibility of Protein and Amino Acids in Hempseed Cake. Asian-Australas. J. Anim. Sci..

[24] Medicine National Academies of Sciences, Engineering, Division on Earth and Life Studies, Board on Agriculture and Natural Resources, Committee on Nutrient Requirements of Dairy Cattle (2021). Nutrient Requirements of Dairy Cattle.

[25] Alfaro-Wisaquillo M.C., Ali M., Patiño D., Oviedo-Rondon E.O., Vann R., Joseph M. (2024). Variations in Soybean Nutritional and Anti-Nutritional Quality Based on Location and Planting Dates. Int. J. Food Sci. Technol..

[26] Heuzé V., Tran G., Nozière P., Lessire M., Lebas F. (2017). Soybean Seeds. Feedipedia, a Programme by INRAE, CIRAD, AFZ and FAO. https://www.feedipedia.org/node/42.

[27] Nowak W., Michalak S., Wylegała S. (2005). In Situ Evaluation of Ruminal Degradability and Intestinal Digestibility of Extruded Soybeans. Czech J. Anim. Sci..

[28] Van Soest P.J., Robertson J.B., Lewis B.A. (1991). Methods for Dietary Fiber, Neutral Detergent Fiber, and Nonstarch Polysaccharides in Relation to Animal Nutrition. J. Dairy Sci..

[29] Qiao G.H., Wang M.H., Lu F., Yang X.F., Xie T.M., Li Z., Li J.X., Sami R. (2026). Diets Including Combinations of Soybean Meal and Urea Effect Rumen Fermentation, Nutrient Digestibility, and Nitrogen Utilisation in Dairy Cows. Anim. Prod. Sci..

[30] Kohli V., Singha S. (2024). Protein Digestibility of Soybean: How Processing Affects Seed Structure, Protein and Non-Protein Components. Discov. Food.

[31] Micek P., Słota K., Górka P. (2020). Effect of Heat Treatment and Heat Treatment in Combination with Lignosulfonate on in Situ Rumen Degradability of Canola Cake Crude Protein, Lysine, and Methionine. Can. J. Anim. Sci..

[32] Kowalski Z.M., Marszałek A., Mills C.R. (1997). The Use of Ca Salts of Rape Seed Fatty Acids to Protect Protein against Degradation in the Rumen. Anim. Feed Sci. Technol..

[33] Masoero F., Fiorentini L., Rossi F., Piva A. (1994). Determination of Nitrogen Intestinal Digestibility in Ruminants. Anim. Feed Sci. Technol..

[34] Van Straalen W.M., Tamminga S. (1990). Protein degradation of ruminant diets. Feedstuff Evaluation.

[35] Rossi F., Fiorentini L., Masoero F., Piva G. (1999). Effect of Fat Coating on Rumen Degradation and Intestinal Digestibility of Soybean Meal. Anim. Feed Sci. Technol..

[36] Alizadeh B., Salamatdoost-Nobar R., Paya H., Parsaeimehr K. (2016). Determination of Degradability of Germinated and Heated Soybean Seeds and Its Proteins Fractions. J. Biosci. Biotechnol..

[37] Busanello M., Velho J.P., Augusto A., Tambara C., Regina D., Alessio M., Neto A.T., Pereira Haygert-Velho I.M. (2016). In Situ Ruminal Degradability of Soybean Meal and Alternative Protein Feeds in Brazil—A Meta-Analysis. Asian J. Agric. Food Sci..

